# Lessons From 3 Longitudinal Sensor-Based Human Behavior Assessment Field Studies and an Approach to Support Stakeholder Management: Content Analysis

**DOI:** 10.2196/50461

**Published:** 2024-10-31

**Authors:** Johanna Kallio, Atte Kinnula, Satu-Marja Mäkelä, Sari Järvinen, Pauli Räsänen, Simo Hosio, Miguel Bordallo López

**Affiliations:** 1 VTT Technical Research Centre of Finland Ltd Oulu Finland; 2 Center for Machine Vision and Signal Analysis (CMVS) Faculty of Information Technology and Electrical Engineering University of Oulu Oulu Finland

**Keywords:** field trial, behavioral research, sensor data, machine learning, pervasive technology, stakeholder engagement, qualitative coding, mobile phone

## Abstract

**Background:**

Pervasive technologies are used to investigate various phenomena outside the laboratory setting, providing valuable insights into real-world human behavior and interaction with the environment. However, conducting longitudinal field trials in natural settings remains challenging due to factors such as low recruitment success and high dropout rates due to participation burden or data quality issues with wireless sensing in changing environments.

**Objective:**

This study gathers insights and lessons from 3 real-world longitudinal field studies assessing human behavior and derives factors that impacted their research success. We aim to categorize challenges, observe how they were managed, and offer recommendations for designing and conducting studies involving human participants and pervasive technology in natural settings.

**Methods:**

We developed a qualitative coding framework to categorize and address the unique challenges encountered in real-life studies related to influential factor identification, stakeholder management, data harvesting and management, and analysis and interpretation. We applied inductive reasoning to identify issues and related mitigation actions in 3 separate field studies carried out between 2018 and 2022. These 3 field studies relied on gathering annotated sensor data. The topics involved stress and environmental assessment in an office and a school, collecting self-reports and wrist device and environmental sensor data from 27 participants for 3.5 to 7 months; work activity recognition at a construction site, collecting observations and wearable sensor data from 15 participants for 3 months; and stress recognition in location-independent knowledge work, collecting self-reports and computer use data from 57 participants for 2 to 5 months. Our key extension for the coding framework used a stakeholder identification method to identify the type and role of the involved stakeholder groups, evaluating the nature and degree of their involvement and influence on the field trial success.

**Results:**

Our analysis identifies 17 key lessons related to planning, implementing, and managing a longitudinal, sensor-based field study on human behavior. The findings highlight the importance of recognizing different stakeholder groups, including those not directly involved but whose areas of responsibility are impacted by the study and therefore have the power to influence it. In general, customizing communication strategies to engage stakeholders on their terms and addressing their concerns and expectations is essential, while planning for dropouts, offering incentives for participants, conducting field tests to identify problems, and using tools for quality assurance are relevant for successful outcomes.

**Conclusions:**

Our findings suggest that field trial implementation should include additional effort to clarify the expectations of stakeholders and to communicate with them throughout the process. Our framework provides a structured approach that can be adopted by other researchers in the field, facilitating robust and comparable studies across different contexts. Constantly managing the possible challenges will lead to better success in longitudinal field trials and developing future technology-based solutions.

## Introduction

### Background and Objectives

Human and environmental monitoring in natural settings is essential in behavioral research. Pervasive technologies such as sensors and machine learning enable the unobtrusive assessment of how individuals behave and interact with their natural environment [1]. Application of such technologies has shown great potential for accurately recognizing emotions and activities in laboratory settings [2,3]. However, brief emotional or activity stimuli induced during an experimental protocol may not fully capture the emotional responses or behaviors occurring in real-world situations. Further studies in authentic environments are required to investigate and validate people’s behavior and the feasibility of novel technologies in naturalistic contexts [4]. Therefore, transitioning research from the laboratory to real-life settings is essential, but it poses additional challenges due to the lack of control over contextual factors and human behaviors [5].

Field studies conducted in naturalistic settings are often regarded as more ecologically valid than laboratory studies, providing a more realistic representation of people’s behavior [4]. These studies frequently involve collecting and analyzing data on people and their surroundings over an extended period to understand the interaction between the users, technology, and contexts and evaluate technologies in uncontrolled situations [6]. However, field trials tend to face additional challenges related to people, changing contexts, and new technology, which are often underreported or dispersed across studies.

To address this shortcoming, this work aims to provide insights and lessons on the challenges and their mitigations that impact the success of real-world trials.

This analysis involves 3 independent longitudinal field studies, each focusing on a different aspect of human behavior and environmental monitoring. Although these field studies are not directly related to one another, they collectively contribute to a broader understanding of the challenges and solutions in longitudinal field trials by answering the following research questions:

1. What kinds of unanticipated challenges arise in longitudinal, sensor-based field trials studying human behavior or activity?
2. How can these challenges be managed?

The main contributions of this work are the categorization of the challenges encountered across the 3 longitudinal field studies and recommendations for conducting real-life studies that assess humans and their environment using pervasive technology.

### Challenges in Field Trials Monitoring Humans and the Environment

A field trial involves collecting data from participants and possibly their environment, typically over a long period. The collected data can be used to develop new technologies and interventions to improve human well-being. The main phases in designing and implementing such a study include defining the research question and objectives, identifying and selecting the study population and sample, designing the study, obtaining ethics approval and informed consent, collecting and analyzing data, and interpreting the findings [7].

Data on human behavior, activity, and environmental factors related to human well-being can be collected through various means such as surveys, interviews, observations, and sensing technologies [4]. There are different applicable sensing methods such as wearables, camera- or radar-based technology, virtual sensors (referring to a software program that simulates the functionality of a physical sensor), and ambient environmental sensors [8,9]. Examples of recent real-life studies on sensor-based monitoring varying from 1 to 10 months in duration include mood, stress, and health prediction [10,11]; daily stress detection [12,13]; and thermal comfort and behavior assessment [14]. However, several factors such as a low recruitment or high dropout rate, technical difficulties, data analysis and interpretation, and ethical reflections often challenge these types of field trials.

Although study participants are initially interested in contributing to the study by providing information or using sensor devices, their willingness can wane over time. Ecological momentary assessments (EMAs; self-reported labels), where participants report their feelings, experiences, and behaviors, are a common way of collecting annotations, that is, ground truth labels [15]. Regular and long-lasting EMAs have been reported to suffer from respondent fatigue [16,17]. Some studies have indicated that EMA scheduling and duration may affect perceived participant burden and dropouts [18,19]. On the other hand, a recent meta-analysis [20] suggests that the number of self-assessments does not appear to correlate with compliance or dropout rates; however, financial incentives have been shown to increase the compliance rates across studies, depending on compensation criteria (eg, providing compensation when at least 70% of self-assessments have been completed).

Furthermore, getting participants to wear or otherwise use the sensor devices consistently can be challenging, which can lead to incomplete data [21]. In addition, participants may feel uncomfortable being constantly monitored, and there may be legal or ethical issues in collecting and storing personal data [17,22]. Assessing and mitigating participant and stakeholder influence on field trial success is important. A recent systematic scoping review [23] identified 21 reports discussing stakeholder assessment approaches for planning stages, but approaches for field trials remained ill-established.

The typical technical challenges of wireless sensor deployment have been related to energy consumption (battery life), communication issues, and protecting devices, which may be especially relevant in outdoor settings [9]. However, these challenges also apply to personal devices such as wearables and smartphones. For example, smartphones have become popular for gathering data in field trials, but continuous data collection may quickly drain the battery [24]. Moreover, collecting, managing, and storing large amounts of data can be costly and time-consuming. The data collection methods must be flexible enough to accommodate changes or unexpected events that may occur during the study [25].

Furthermore, real-world studies are susceptible to various sources of bias such as selection bias and measurement bias, which can affect the validity of the study. For instance, machine learning models require ground truth labels against which their accuracy can be compared. EMAs are subject to cognitive bias, as participants may not always be aware of their state, and to social desirability bias, where participants present themselves in a certain way they perceive favorably [26]. External observers can also label behavior, but this can be costly and time-consuming, and the observer may not always interpret the state of the participant correctly.

Interpreting human behavior is difficult due to the unstructured nature of real-world data and the lack of a consistent theoretical background; hence, behavioral modeling is often based on simplified theoretical concepts [27]. Moreover, modeling human activity and behavior is particularly challenging because emotions, expressions, and movements are highly distinctive for every individual [28]. This means that general models may be inaccurate for individual users because they are not tailored to the characteristics of a specific individual and context [29]. While personalized models can be tailored to the characteristics of a particular individual and environment, they may not be well generalizable and thus less widely applicable.

### Frameworks for Field Trials

To tackle challenges related to the capabilities of wireless sensors, Booth et al [25] presented a framework for orchestrating sensor data streams through various pathways in real-life studies. The framework consolidates multimodal sensor data in one backend location, facilitating automatic stream monitoring and participant feedback systems. The authors also discussed criteria and factors for sensor selection and testing, including study site preparations and data collection. Moreover, the authors emphasized the importance of conducting a sensor survey that considers data quality and data access logistics, costs, technical requirements, ethics, and participant engagement before testing and data collection.

The perception and interpretation of context are essential in all interactions between humans or a human and technology [30]. Groh and Picard [31] have developed a conceptual framework for categorizing the context in automated affect detection using 7 factors. An ambient sensory environment refers to sensed information regarding the immediate surroundings. Situational constraints are caused by the activity or the immediate environment [32]. Furthermore, factors related to people, such as social relationships and cultural orientation, are defined as sociocultural dimensions. A temporal dimension refers to the time and dynamics of an event, such as an emotional expression or a specific behavior. Personalization includes all individual characteristics such as demographics, personality traits, abilities, or disabilities [33]. Methods of measurement refer to annotating emotions and human behavior. Furthermore, measuring human emotions and behavior also requires a semantic representation referring to a way of representing the meaning of a state. Overall, the framework developed by Groh and Picard [31] highlights the importance of considering all these contextual factors when detecting emotions and behavior to achieve more accurate results.

## Methods

### Research Process

To answer the research questions, we adopted a case study approach and looked for suitable types of field trials from our previous research using the following criteria: (1) carried out in real-life context, (2) investigated human behavior or activity, (3) were longitudinal with a continuous duration of ≥1 month for the data collection, and (4) used sensors for collecting primary data. After reviewing the field trials, 3 field studies met the criteria and were selected for further analysis—stress and environmental assessment in an office and a school, work activity recognition on a construction site, and stress recognition in location-independent knowledge work. Although these field studies were conducted independently, collectively they provide valuable insights into the challenges and solutions in longitudinal field trials by identifying similarities and differences across diverse contexts.

In the first phase, we developed a qualitative coding framework to systematically code and categorize the challenges faced in each field study. This novel framework was designed to include multiple dimensions of field trials such as influential factor identification, stakeholder management, data harvesting and management, and analysis and interpretation. By integrating and expanding on the existing frameworks, our approach offers a holistic tool that researchers can use to navigate the complexities of longitudinal sensor-based field trials. We used inductive reasoning to analyze written documentation of the selected field studies, including data management plans, technical reports, and internal memoranda from weekly meetings stored in project archives. The primary objective was to identify unexpected difficulties faced during the studies, mitigation actions to address the challenges, and lessons for future studies. Inductive reasoning allowed us to identify prominent, frequent, or substantial themes from the data without constraints from structured methods [34].

In the second phase, we synthesized the findings to develop a comprehensive understanding of the problems encountered and lessons learned. The synthesis enabled us to compare the types and extent of challenges across the field studies, providing valuable insights into their similarities and differences. In the third phase, we adopted methods proposed by Ballejos and Montagna [35] to identify and evaluate the influence of different stakeholders on the success of the field study. Analysis was done by researchers directly involved in the field studies, using the coding framework to ensure comparability. The findings were triangulated by having the researchers first do the analysis separately and then jointly review the findings to ensure consistency in interpretation. Finally, the most important findings were summarized as recommendations provided in the discussion section of this paper.

### Coding Framework

We used 2 established frameworks that categorize themes noted in challenges in field trials for human and environmental monitoring as a starting point for the coding framework: one by Booth et al [25] that pertains to sensor selection, deployment, and management and the other by Groh and Picard [31] that focuses on affect recognition. We also evaluated the challenges and recommendations reported in other studies (eg, L’Hommedieu et al [17]) to further identify the key categories that must be considered when planning and conducting these trials. On the basis of the existing frameworks, reported challenges, and our own experiences, we identified 4 main categories to consider when designing and conducting field trials: influential factor identification, stakeholder management, data harvesting and management, and analysis and interpretation.

Influential factor identification is essential to understand the background factors that may impact the success of a trial. For this category, we identified two key themes that should be considered during the planning and execution phases: (1) phenomenon and (2) context and duration. Phenomenon refers to the specific human behavior or activity being monitored and the characteristics of this phenomenon that are relevant to the trial [31]. Context refers to the environmental, sociocultural, and situational factors that can impact the success of a trial, such as physical settings, organizational norms and resources, and social interaction [31,32]. Duration refers to the measurement period and its potential impact on the trial.

Stakeholder management is critical for the success of longitudinal field trials involving human and environmental monitoring [17]. This category covers (1) participant engagement, (2) partner management, and (3) stakeholder engagement. Participant engagement involves recruiting and engaging individuals or groups whose data are being collected during the trial, including obtaining informed consent for data collection and use [17]. Partner management refers to collaborating with other organizations or individuals involved in the trial, such as academic institutions or research groups. Finally, other stakeholder engagement refers to the engagement of stakeholders who are not participants or partners but have a vested interest in the trial’s execution or outcome, such as the management of the trial organization, local communities, government agencies, and other organizations the trial may impact.

Data harvesting and management is critical to any field trial using sensor technologies. This category builds mainly upon the work of Booth et al [25] and covers three essential themes: (1) measurements and annotations, (2) data transmission and connectivity, and (3) data storage and management. Measurements and annotations involve using sensor technologies to gather data during the trial and annotations or additional information that may be required to understand the data [31]. Data transmission and connectivity is needed to connect data sources and transfer data from the trial location to a central repository, while also considering data security and privacy [36]. Data storage and management includes data backup, access and control, and archiving [37].

Analysis and interpretation includes (1) data validation and (2) model development and validation. Data validation evaluates data quality, including statistical and computational bias [38]. Model development relates to the selection and use of machine learning and statistical methods for feature extraction and algorithm development [31]. Model validation includes approaches for explainability and techniques for model validation, including the identification of influencing factors for analysis.

### Ethical Considerations

In all cases, the study plan included considerations regarding accountability, fairness, and responsible communication to promote freedom of choice and avoid potential harm when conducting field studies using novel sensing technology. The need for ethical permissions was evaluated by the Ethics Committee of VTT Technical Research Centre of Finland Ltd (henceforth VTT) against the guidelines from the Finnish National Board on Research Integrity [39]. In case A, case B, and the second pilot of case C, specific ethical permission was not required. However, permission was needed for the first pilot of case C, and the Ethics Committee of Hospital District of Helsinki and Uusimaa approved it (HUS/2536/220).

In all field studies, informed consent was obtained according to the European research integrity and General Data Protection Regulation (GDPR) guidelines, including for research objectives, study process and duration, benefits and potential disadvantages of the research, bodily integrity of the participant, voluntariness and withdrawal from the study, publishing of the research results, data collection, processing and archiving, data subject’s rights, and contact information. The consent states that the data collected will be used for research that extends beyond the current projects, allowing secondary analysis without requiring a new consent.

The studies followed the GDPR in setting up data protection measures. Only data relevant to the research questions were collected. Data were pseudonymized during collection and anonymized at the time of archiving. Data are locally stored, protected by firewall, and accessible only by the researchers involved. We also adhered to other GDPR requirements such as data remaining within the boundaries of the European Union.

In the first field study, no compensation was provided to the human participants. A set of workwear was provided in the second field study. In the first pilot in the third case, personal analysis of the physiological data was provided after the pilot, and a gamification approach was used in the second pilot where participants were offered small snacks as rewards based on their reporting activity. The information contained in this paper does not contain any identifiers of participants in the 3 field studies.

### Empirical Data

This section presents the 3 field studies selected for further analysis. Certain regulatory restrictions and ethical requirements were common to all field studies deploying sensors and cloud platform–based measurements in real-life settings.

#### Case A: Stress and Indoor Environmental Quality Assessment Using Environmental Sensor Data

Work stress is a complex phenomenon influenced by various psychosocial and physical factors, including poor environmental conditions. Exposure to environmental stressors, such as elevated carbon dioxide levels, has been shown to impair cognitive performance [40] and cause discomfort [41]. Thus, one of our field studies focused on stress detection, especially related to environmentally induced stress, and how to mitigate risk factors to improve well-being. We conducted 2 long-term indoor environmental quality (IEQ) data collections at school and office environments to study the applicability of IEQ sensor data for continuous stress and IEQ assessment of employees during the fall of 2018 and spring of 2019 [42].

The field study lasted for 3.5 months at the school and 12 months at the office facility. We recruited participants from an elementary school and a knowledge work organization in Northern Finland. The inclusion criteria for research participants were as follows: (1) uses an Android phone for self-reporting, (2) commits to wearing a wearable device during the workday, and (3) commits to answering self-reports daily. There were 27 voluntary and eligible research participants: 4 teachers and 23 office workers. The mean age for teachers was 43.5 (SD 15.6) years, and for the office workers, it was 41.6 (SD 8.0) years. All teachers (4/4, 100%) and 48% (11/23) of the office workers were women. All participants gave written informed consent.

While planning the monitoring of teachers, knowledge workers, and their environment, we engaged with several stakeholders, including the facility owners, elementary school principal, research management, and human resource (HR) departments. These stakeholders agreed upon the installation and use of a continuous monitoring system. In the elementary school setting, proper consideration to what could be measured in the presence of susceptible individuals, that is, children, was essential. Guardians of the schoolchildren were also informed about the measurements, but no data were collected directly from the schoolchildren.

Regarding data harvesting, we collected diverse data streams with varying levels of granularity to produce representative data for modeling, including annotations. The teachers reported their perceptions of IEQ, stress, and productivity on weekdays for 3.5 months and the office workers reported the same for 3.5 to 7 months via an Android self-reporting app developed by us. In addition, 2 time-triggered quantitative questionnaires on perceived IEQ, stress level, and productivity were scheduled daily, in the mornings and afternoons. Moreover, the research participants wore a wrist device to monitor physiological data (continuous activity and heart rate) during working hours.

The sensor data were gathered with a set of customized sensor devices integrated in a TinyNode hardware platform developed by VTT. The hardware platform included commercially available sensors to measure temperature (in °C), relative humidity (calculated in %), air pressure (in hPa), carbon dioxide (in ppm), and activity level based on passive infrared sensors (sum of detected motion events; 0-12). The passive infrared sensor data reflected movement for each seating place. The TinyNode operates on a cell battery and communicates wirelessly over Bluetooth Low Energy. Data samples were obtained once per minute for all parameters. The positioning of IEQ sensor devices followed the national legislative recommendations [43]. The gateway devices in school study rooms used a commercial router with 4G mobile network capabilities for internet access, while the gateway devices in the office rooms used the available wireless or wired internet connections. The gateways run on a Raspberry Pi single-board computer, and data were sent to the Microsoft Azure cloud platform via a message queuing telemetry transport protocol over transmission control protocol/IP. Data retrieval from Azure used a published application programming interface for Microsoft Azure TableStorage.

For IEQ, stress, and productivity analysis, we performed feature selection, classification model training, and evaluation separately for each research participant due to the dependence of IEQ, stress, and productivity interpretation on individual perceptions. Time windows of predetermined length were used to extract features with the Python library “tsfresh.” Feature selection involved calculating correlations between extracted feature values and respective self-reports. A support vector machine algorithm was used for classification.

#### Case B: Construction Site Safety Monitoring With Internet of Things Sensors

The labor-intensive construction industry has many occupational health and safety challenges such as accidents and nonergonomic work conditions [44]. Recently, there has been a shift toward using sensor-based and data-driven solutions to improve safety on construction sites [39]. In 2020, we conducted a field study to evaluate the potential of these solutions. Our study used a Bluetooth-based positioning system and inertial sensors to identify safe and potentially unsafe activities of workers in laboratory and field settings [45].

The case was implemented on a construction site in the capital region of Finland for 15 weeks. Site access was limited for safety reasons and to minimize disruptions to the work. Initially, 15 construction workers with different occupations such as painters; heating, ventilation, and air conditioning mechanics; and cleaners were recruited to participate in the field study. The inclusion criteria were that participants should be of working age and employed full time, but detailed demographic data were not collected because they were not purposeful for the study. All participants provided written informed consent. Two research consortium partners provided material for the field study: the workwear with special sensor pockets and the Internet of Things (IoT) sensor units. Other stakeholders included the subcontractor companies of the recruited workers because 11 out of 15 study participants were subcontracted workers on the construction site. In addition, the trade union for construction workers was recognized as an influencing stakeholder, and active discussions and a survey on new technologies for construction site safety were carried out with them.

For the activity data collection, the construction workers wore smart workwear with 3 special sensor pockets and 3 sensors for 4 days a week, from Tuesdays to Fridays. Mondays were reserved for sensor maintenance, including data download for storage, battery replacement, and checking sensor operational status. Two of the sensors were IoT units primarily for motion detection, and one sensor was for positioning based on Bluetooth technology. The IoT units were 10-axis inertial measurement units composed of a 3-axis accelerometer, a 3-axis gyroscope, a 3-axis magnetometer, and a barometer, with fixed sampling rates of 103 Hz for the accelerometer, 97 Hz for the gyro and magnetometer, and 0.033 Hz for the barometer. IoT unit data were saved on the on-device flash memory. On Tuesday mornings, the construction workers fetched the sensors from a locker in the personnel room and placed them in the correct pockets and in the right orientation. They were expected to return the sensors to the locker every Friday for maintenance. The IoT sensor platform used in the study was a new version of a commercial product and had not gone through extensive testing. The positioning service was a commercial product, and the data were accessed via a web service.

The data collection system running on the construction site generated large amounts of unlabeled data with varying quality. The collected data were analyzed and interpreted using the activity recognition models created with laboratory-collected training data. Although the laboratory results could not be verified in the field study, they showed promising results that wearable IoT sensor data and machine learning could be applied to recognize different activities and provide valuable information about different work activities, working poses, and phases on construction sites.

#### Case C: Work Stress Detection Using a Computer, a Keyboard, and Mouse Dynamics

A quarter of the European Union employees have experienced frequent or constant stress at work [46]; thus, feasible and validated solutions are needed to detect early signs of prolonged work stress and support occupational well-being. Analyzing behavioral data is a way to detect stress as stress is manifested in human behavior [47]. Analyzing computer use, that is, digital behavior, is a potential approach, but previous studies with this approach have been conducted in laboratory conditions or in contexts where tasks are very uniform, such as student examinations [48]. We studied the applicability of computer use data for stress monitoring in knowledge work by conducting long-term field experiments in 2 organizations in 2021 and 2022.

The study was carried out in 2 pilots lasting 2 to 5 months. The first pilot had 38 participants from 2 knowledge work organizations, and the second pilot had 19 participants from 3 organizations. The mean age for the participants in the first pilot was 44.5 (SD 9.9) years, and 82% (31/38) were women. The mean age for second pilot participants was 38.4 (SD 11.6) years, and 53% (10/19) were women. The inclusion criteria for the participants were that they (1) were working regularly and (2) had a work contract covering the study period, and for the second pilot, (3) they had a fluent understanding of English. Recruitment was carried out locally in the organizations, but participants had a central point of contact during the study. The exclusion criterion was acute health conditions. All participants provided written informed consent.

Apart from the study participants, several other key stakeholders were involved. Another research organization was involved in the study, HR teams were engaged in recruiting participants, and deploying monitoring software in several separate organizations required negotiations with the respective IT or security teams.

Data were harvested using a virtual sensor, that is, a monitoring software installed on the work computers. The virtual sensor generated logs of mouse trajectories, keyboard typing tempo, and application switching and sent the data daily to the cloud backend. Actual keystrokes were not logged, except for Del and Backspace, for content security reasons. Contents of the screen were not captured. Applications were grouped into 5 broad categories, namely communication (eg, Microsoft Teams and Skype); web (all browsers); documents (eg, Microsoft Word and Microsoft PowerPoint); other (everything not falling into the above groups, such as software development tools); and unknown (rare cases when the operating system did not or could not return application information, such as system-level tasks). We did not gather data about application content, such as websites, or other computer use data, such as video or voice. The original data collection plan also included motion and IEQ sensors.

The data labels were collected via a self-report application provided for Windows, Android, and iOS platforms, which sent the daily reports to the cloud backend. Participants reported their perceived stress levels (stress, calm, and positive stress); work content (simple, need to do, interesting, and challenging); skills needed (little, average, lots, and need learning); productivity (on a 3-level scale); and optional work-related reasons for experienced stress from Monday to Friday. On Fridays, participants also answered an additional question on the overall stress level of the work week. The backend was located on the Microsoft Azure platform, accessible through a transmission control protocol/IP connection. Authentication for both virtual sensor and self-reporting data was done with the OAuth2 method. No actual names, IP addresses, or other identifiers were recorded in the transfer process. Data retrieval from Azure uses a published MySQL application programming interface for Microsoft Azure TableStorage. The backend provides role-based access control to data, with data encrypted when at rest, to minimize risks if data security was breached.

The analysis phase was ongoing at the time of writing this paper. The analysis started during the data harvesting phase, using data from the first participants, with more data added as each next batch of participants finished their data collection. Various feature extraction, feature selection, and classification methods are being tested using the research organization’s internal computing servers.

## Results

### Identified Challenges From Field Studies

The analysis results from applying the coding framework themes to the selected field studies are presented in Tables 1-3. The tables present the challenges and actions carried out during the project to counter the challenge (ie, mitigations) or lessons learned during the project for future studies.

**Table 1:** Challenges identified in case A: stress and indoor environmental quality (IEQ) assessment using environmental sensor data.

Theme		Challenges and possible mitigation measures or lessons
**Phenomenon**		
	Challenge	Showing the causality between IEQ, stress and productivity in natural settings with a small set of labels
	Mitigation	Lowering the target level to find associations instead of causality was more feasible within the schedule and resources of the study
**Context and duration**		
	Challenge	Labeling duration varied from 3.5 to 7 months and did not cover all seasons, yet, seasonal variation has a substantial impact on IEQ in the region
	Lesson	Reflect if seasonal variation influences the phenomenon measured
	Challenge	Relocations in the office due to unexpected facility renovation caused additional work and loss of participants and data
	Lesson	Consult in advance with the stakeholders regarding potential changes in the study environment and schedule; explore alternative study locations
**Participant engagement**		
	Challenge	Difficult to enroll participants; many potential participants were unwilling to commit to 4-6 months of measurements
	Lesson	Use incentives (material or nonmaterial, such as personalized analysis reports) to engage participants and reduce dropouts
	Challenge	A dropout rate of 30% due to loss of interest or relocation at the office to a location not allowing sensors
	Lesson	Prepare for potential dropouts when planning
	Challenge	Some participants expected that the data would validate their perceived symptoms, such as fatigue, and reveal the stress factors, leading to unrealistic expectations of the results
	Mitigation	An additional information session was organized
	Challenge	The number of self-reports decreased because of personal restrictions such as hurry, holidays, and business trips
	Lesson	Offer incentives to increase compliance rates and simplify self-reporting as much as possible
Partner management		No challenges were identified from the data for this category
**Other stakeholder engagement**		
	Challenge	Some other stakeholders had unrealistic expectations for the study results; for instance, facility owners wanted the data to confirm that the facility did not have air quality problems
	Mitigation	An additional information session was organized
**Measurements and annotations**		
	Challenge	Android app alerts failed for some participants, possibly due to variations in operation system versions
	Lessons	Recognize common mobile app issues; providing a web application and using calendar reminders can help; monitor data accumulation to detect problems
	Challenge	The wearable device to measure heart rate was not worn regularly or tightened enough, leading to inconsistent use and loss of data
	Lesson	Monitor data quality and provide instructions; offer incentives for compliance
**Data transmission and connectivity**		
	Challenge	Data loss (total of 24 hours) due to interruptions in internet connectivity and problems reconnecting to the Azure database
	Mitigation	A fault recovery system for database connection solved this issue
	Challenge	Electromagnetic interference caused a loss of 0.5%-1.5% of Bluetooth packets per day in most offices
	Lesson	Estimate possible data loss when defining the target for the amount of data
	Challenge	Long-term jitter in the timer of the sensor firmware and the lack of intersystem synchronization causing missing samples
	Lesson	Perform a sufficiently long field test to detect possible data loss
**Data storage and management**		
	Challenge	Relying on external commercial cloud platforms for backend operation for 3.5 years resulted in relatively high final costs
	Lesson	Prepare for data storage expenses in the budget
**Data validation**		
	Challenge	The physiological data were unusable due to quality problems (motion artifacts), although the participants were carefully instructed on how to wear the wearable device
	Lesson	Test beforehand, monitor data quality, and provide instructions
	Challenge	Out of the 23 participants, data from only 15 (65%) were eligible for analysis due to lost sensor data and incomplete self-reports
	Lesson	Prepare for data quality issues by recruiting more participants and monitoring constantly
**Model development and validation**		
	Challenge	The participants reported daily work stress, productivity, and IEQ perceptions on a 5-level scale, but the limited number of reports made it impossible to classify the responses into 5 categories
	Mitigation	With an average of around 75 self-reports per participant, binary classification models were possible to train
	Challenge	As the data collection activities required more resources than expected, testing of multiple model development approaches was not possible, potentially leading to suboptimal results; models were tailored closely to participants, possibly reducing general applicability
	Lesson	Allocate resources for analyses

**Table 2:** Challenges identified in case B: construction site safety monitoring with Internet of Things (IoT) sensors.

Theme			Challenges and possible mitigation measures or lessons
**Phenomenon**			
	Challenge	Identifying and modeling motion features leading to accidents and safety issues, which are short term and rare	
	Mitigation	Changing the modeling target toward nonergonomic poses and accident-prone working activities (eg, climbing a ladder) rather than motions	
**Context and duration**			
	Challenge	Participants left the study because they did not work on the same construction site for 15 weeks	
	Lesson	Consult beforehand with stakeholders regarding the ways of working and schedule	
	Challenge	The construction site was a constantly changing environment posing challenges to maintaining the positioning sensor infrastructure; the Bluetooth anchors were removed without informing the researchers when the anchors prevented the work (eg, painting the wall where the anchors were attached)	
	Lesson	Consult beforehand with stakeholders regarding potential changes in the environment	
	Challenge	Safety and security policy prohibited researchers’ free access to the construction site; researchers felt that their visits and communication were a nuisance for everyday work on-site	
	Mitigation	Minimizing the number of visits and planning carefully to maximize their value	
**Participant engagement**			
	Challenge	Recruitment went through the site manager (no direct contact with participants) without visibility to the subcontractor chain; researchers met the participants only twice, once in the selection of needed workwear and then in the study kick-off delivering the workwear and explaining the study protocol; this took place in a coffee room where it was difficult to understand who the actual participants were	
	Lesson	Organize a dedicated session only with participants	
	Challenge	Many dropouts due to workers leaving the site and the study protocol being too tedious; researchers were not informed about the dates when the participants left the site, but data showed the first 5 weeks to be active in data collection.; after 6 weeks, there were only 7 participants left	
	Lesson	Prepare for dropouts by recruiting more participants; offer incentives	
	Challenge	Participants forgot to fetch or return the sensor, causing problems with data collection	
	Mitigation	Clear instructions were given to participants, also attached by the locker where sensors were stored, and the site manager was asked to remind the study participants; explicit study material provided for the participants’ employers (including subcontractors)	
**Partner management**			
	Challenge	Workwear provider wanted to promote their offering, causing issues in clothing selection and delivery; the technology setup was under development, and the number of sensors integrated to the workwear was not fully clear to the provider in the planning phase	
	Mitigation	Researchers actively discussed, informed, and documented the study planning, and the material was available on the research project’s document platform	
Other stakeholder engagement			No challenges were identified from the data for this category
**Measurements and annotations**			
	Challenge	To minimize disruptions to work, the workers could not be asked to annotate their daily work; over a 15-week period, observation was limited to following 3 workers on two separate occasions for 2 hours each; this severely limited the amount of labeled data and challenged the subsequent model development	
	Lesson	Understand and prepare for the limitations due to the environment	
	Challenge	Due to study requirements, a higher data sampling rate for position data was used than the commercial system was designed for, leading to unexpected technical problems and unusable data	
	Lesson	Perform a sufficiently long field test to detect issues	
	Challenge	In some cases, especially when the participants forgot to return the sensors, the IoT sensor units ran out of battery, leading to data loss	
	Mitigation	The site manager was asked to remind participants	
**Data transmission and connectivity**			
	Challenge	The IoT sensor units collected data to an internal memory card, which failed if the battery ran out, corrupting the memory card and causing the loss of all data; also, if the sensor memory was not erased, it caused problems for the following week’s data collection	
	Mitigation	A firmware update was created and applied to the IoT units	
	Challenge	Data transfer to storage was a manual and complicated act, where even a slightest mistake could lead to data being erased and lost	
	Lesson	Prepare for problems with experimental setups. Avoid using experimental sensors in data collection for artificial intelligence development	
Data storage and management			No challenges were identified from the data for this category
**Data validation**			
	Challenge	Location data could not be used due to quality issues (see the Measurement and annotations category in this table); data gaps also existed in the IoT data (see the Data transmission and connectivity and Measurements and annotations categories in this table); a drift in sensor timestamping was noticed during the study period, which also caused validation problems	
	Mitigation	To minimize drifting, postprocessing of timestamps and alignment of signals was performed	
**Model development and validation**			
	Challenge	Validation of data analysis results was difficult due to the minimal number of annotated data samples	
	Mitigation	A separate data collection exercise in the laboratory mimicking the real world was performed to support model development; however, using models based on laboratory data was challenging due to the diversity of work activities performed in real sites; another data annotation (observation) period was organized at the end of the field study	

**Table 3:** Challenges identified in case C: work stress detection using computer, keyboard, and mouse dynamics.

Theme		Challenges and possible mitigation measures or lessons
**Phenomenon**		
	Challenge	Stress is a physiological but also partially subjective phenomenon. There is no consistent and accurate method to establish ground truth for stress
	Mitigation	Research used self-reports as the ground truth
**Context and duration**		
	Challenge	The COVID-19 pandemic prevented studies according to initial plans. Research on environmental stressors had to be dropped
	Mitigation	A context-independent virtual sensor to measure computer use behavior
	Challenge	Study organization had security-critical projects, and IT saw the use of the virtual sensor (keylogger technology) as too risky
	Mitigation	An external security audit of the virtual sensor
	Challenge	A loss of technical know-how due to a researcher leaving the project led to a mistake, risking a loss of a substantial amount of data; significant manual effort was required to recover the data
	Lesson	At least 2 persons should be familiar with the technical setup and the setup should be carefully documented
**Participant engagement**		
	Challenge	Recruitment was significantly less successful than anticipated; enough participants were eventually recruited, but required extra effort and complicated the study setup
	Mitigation	Continued recruitment and extending the recruitment to other organizations; the field study ran the pilot in “waves” until the recruitment target was met
	Challenge	Commonplace practical challenges, such as people not reading the instructions, or losing or forgetting their passwords, causing additional workload to the research team
	Mitigation	Increased communication and support effort, especially at the early stages of the measurement for the second pilot study
**Partner management**		
	Challenge	Cross-organizational study coordination and achieving mutual understanding for fluent cooperation in a cross-disciplinary (behavioral science, data science, and information and communication technology professionals) group was challenging and required more communication effort than anticipated
	Mitigation	Additional communication and joint weekly meetings
**Other stakeholder engagement**		
	Challenge	Research organization’s DPO^a^ had GDPR^b^ compliance concerns for voluntariness in recruitment and data sensitivity; negotiations with DPO delayed the project start, and agreed measures increased study management complexity and caused extra costs
	Mitigation	Arrangements to ensure participation anonymity, additional data security measures to the backend
	Challenge	The field study organization’s IT and security team considered the virtual sensor a security risk; providing documentation instead of having direct discussions was a mistake, resulting in an initial prohibition and an additional 5 months of negotiations, leading to a delay and unanticipated costs
	Lesson	Actively engage with IT and security teams to get approval already when planning to use tools that may cross their domain
	Challenge	Candidate study organizations were asked to test technical solutions very late in the study planning cycle; one candidate organization withdrew from the study due to technical problems that were discovered too close to the start
	Lesson	Perform operative testing early enough to identify technical problems
**Measurements and annotations**		
	Challenge	Physiological sensors were considered unreliable ground truth due to motion artifacts; self-reports of stress were feasible but subjective and prone to lapses
	Mitigation	Self-reports to be treated as ground truth; gamification used to entice people to keep reporting
**Data transmission and connectivity**		
	Challenge	Automated data harvesting application authentication failed at times, requiring repeated reauthentication from users, causing frustration among participants and added workload for the technical staff due to password reset requests
	Lesson	Develop a streamlined method for reauthentication (such as browsers’ password storage feature) when using digital reporting tools
	Challenge	Data transmission had to be tailored for each study organization’s information system network, causing deployment delays
	Lesson	Allocate budget and time for technical implementation
**Data storage and management**		
	Challenge	Locally developed solutions required active maintenance and specialized knowledge, increasing technical workload and budget use beyond estimated (25% of the effort budget)
	Lesson	Use commercial options when available or allocate substantial budget and time for technical implementation
	Challenge	Relying on external, commercial cloud platforms for backend operation carries relatively high final costs
	Mitigation	Sufficient budget was allocated for cloud expenses
**Data validation**		
	Challenge	Features from prior art fail to correlate well with ground truth labels, possibly due to it being derived from short (few hours maximum) measurements typically done in a laboratory context
	Mitigation	Extending the project duration to allow more experimentation
	Challenge	Stress behavior can vary significantly from one person to another; features that work for one person do not necessarily work for another (no universal feature); limited number of labels does not allow reliable feature selection based on labeling
	Mitigation	Extending the project duration to allow experiments with different features
Model development and validation		No challenges to report at the time of writing this paper

^a^DPO: data protection officer.

^b^GDPR: General Data Protection Regulation.

### Summary of the Case Findings

The case-specific findings demonstrated various challenges relevant to all viewpoints in the established coding structure, some of which were mitigated during the project, while others were taken as lessons for future projects. Some phenomena are difficult to detect in real-world settings, and longitudinal studies are likely to face changes in the environment and conditions in the field. Consulting stakeholders to understand what changes might happen is critical, but some things cannot be fully anticipated, and the ability to adjust is needed. Problematic changes can also occur in the research team itself, and the more dependent the field study is on a person’s expertise, the more critical it is to document carefully and have another person who can take over.

Stakeholders, including study participants and collaborators, constitute another major issue area. Achieving a common understanding can be challenging; there may be needs, interests, and expectations that the study is not intended or is unable to meet or may even conflict with, and the interest may wane over time. A particularly challenging case is dealing with stakeholders with influence but without direct interface with the research team. Communication is the key in this respect—frequent and clear, with an initiative to discuss issues as early as possible. Another essential tool is to find incentives that help to maintain the support for the study.

While pervasive technology is a significant enabler for conducting field studies on human behavior, it also presents many problems. The reliability of the technology in the field is rarely 100%, and environment or even personnel changes can cause additional issues. The outcome is often data loss, which in turn causes problems in data analysis and interpretation. Technology trials in realistic settings before deployment, designs that include fault recovery systems, and monitoring data accumulation and quality are important means that help. However, some data loss or data quality issues are likely unavoidable in field trials; therefore, extending the study duration beyond what would be needed if everything goes perfectly is necessary to ensure enough data for analysis.

### Stakeholder Mapping

**Figure 1:**
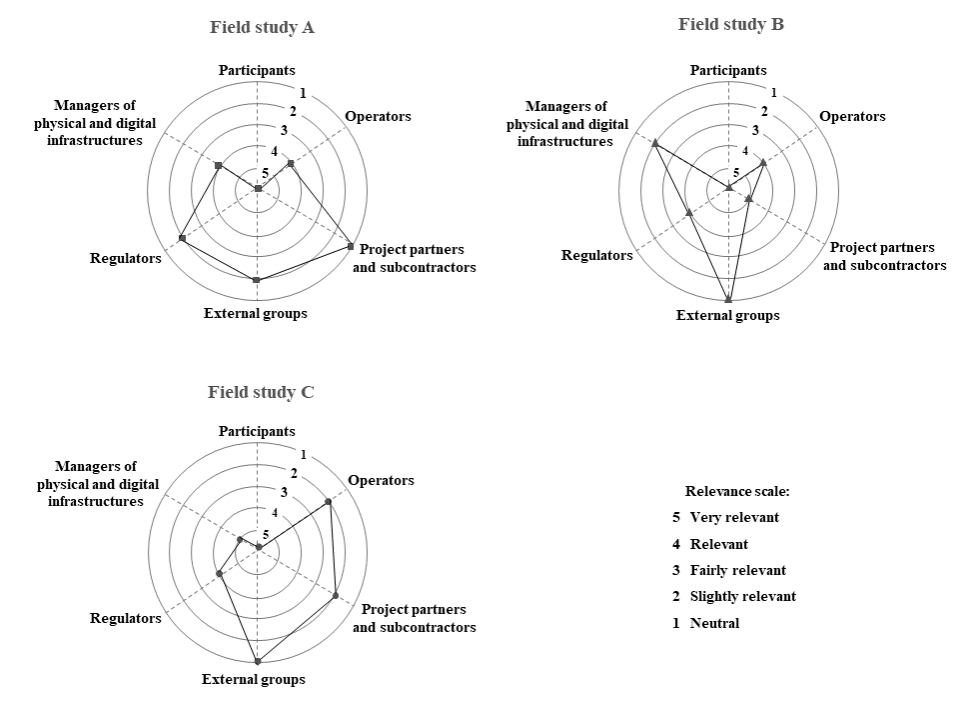
Stakeholder mapping in field studies: (A) stress and indoor environmental quality assessment using environmental sensor data; (B) construction site safety monitoring with Internet of Things sensors; and (C) work stress detection using a computer, keyboard, and mouse dynamics.

In the analysis phase, the importance of stakeholders and their management was raised as a significant issue as a key enabler for the study’s success and a frequent source of challenges. Therefore, we analyzed this aspect further, as there was evident variation in the related challenges and roles, as well as similarities among the stakeholders. This resulted in identifying the main stakeholder groups and allowed mapping out their relevance and importance in the field studies, as presented in Figure 1. Most of these groups were present in all 3 field studies. The key difference in importance in our mapping was their impact on the study success, with those that can stop or enable the study, and even influence the data quality, thus having a major impact on study success being in the center, and those that can hinder or support the study being in the outer area.

The primary stakeholder group includes the *participants of the study*, as the essential data are harvested from them. The next key group is the *operators* of the study environment or their representatives, such as participants’ employers or their HR departments. Next are the *project partners and their potential subcontractors*, including service providers that the project uses to assist in a particular aspect of the study, for instance, positioning service or cloud platform facilities. The third and fourth groups are the *managers of the physical and digital infrastructures* in the study context, for instance, office facility management and IT and security departments. The fifth important group is the *regulators*, as requirements, for instance, those governing safety issues at construction site or dictating how IEQ measurement needs to be done, or through roles, such as the data protection officer for GDPR issues. The sixth and final group is the *external groups* with a vested interest, especially toward the research participants, for instance, parents of the children at school or trade unions, who can influence the study either by supporting or hindering it.

## Discussion

### Summary of Findings of the Specific Field Studies

#### Overview

Transitioning research from the laboratory to the real-world setting brings significant challenges, and the success of a study depends on overcoming them. This study aimed to provide new information on what challenges researchers will likely face when conducting sensor-based field trials over a continuous longitudinal duration and how those challenges could be managed. To answer our research questions regarding the unpredictable challenges faced in sensor-based field trials studying human behavior or activity and possible solutions, we analyzed and synthesized the challenges and lessons of 3 longitudinal field studies.

Each independent field study highlighted specific lessons that can inform future research and practical applications in similar settings.

#### Case A: Stress and IEQ Assessment

Case findings revealed that aiming for associations rather than causality might be more feasible within the constraints of such studies. Seasonal variations impact IEQ and stress, underscoring the need to account for these factors in longitudinal field research. Effective communication with stakeholders about potential changes and realistic outcomes is essential. Offering incentives can improve participant compliance and reduce dropouts. Monitoring data quality and addressing technical issues promptly can mitigate data loss.

#### Case B: Construction Site Safety Monitoring With IoT Sensors

In the dynamic environment of a construction site, modeling should focus on broader activity categories such as nonergonomic poses and accident-prone activities. Direct participant interaction and incentives can improve engagement and data quality. The operator on-site needs to commit to actively support the study implementation. Pretesting experimental setups thoroughly is necessary to minimize technical issues during the study. Robust methods for data annotation should supplement limited observation opportunities.

#### Case C: Work Stress Detection Using Computer Keyboard and Mouse Dynamics

Given the subjective nature of stress, self-reports were used as ground truth data. The study demonstrated the importance of flexibility in adapting to unforeseen events such as the COVID-19 pandemic. Early engagement with IT and security teams is crucial to address concerns and ensure compliance. Maintaining thorough documentation and cross-training team members can mitigate risks associated with personnel changes. Continuous recruitment and extended study periods are necessary to meet participant targets and ensure sufficient data collection.

### Principal Findings and Comparison With Prior Work

The findings of case studies demonstrated challenges in 4 categories: *influential factor identification, data harvesting and management, stakeholder management,* and *analysis and interpretation*.

Concerning influential factor identification, sensor-based monitoring of humans and their environments requires understanding the phenomenon under investigation [27], as typically established through previous research and applied to define the main study objective and methods. In many cases, the environment changes due to explicit human operation or implicit use of the space (eg, different modes in office work). The need to tailor the protocols and solutions for the specific context and build in flexibility to adapt to a changing environment is standard when conducting research in a natural setting and should be reflected in the schedule and budget.

In sensor data harvesting and management, we faced technical issues with wireless connectivity and data transmission despite our testing efforts. This highlights the importance of conducting operative testing early and for a sufficient duration to detect technical problems, as well as implementing an automatic fault recovery system. In general, deploying pervasive solutions increases the scale of data harvesting and management, which means that technology setups require IT and software expertise, as well as maintenance work. Furthermore, behavior data analysis becomes intractable without annotations. Although making daily self-assessments with reminders effortless for the participants, case 1 suffered respondent fatigue without incentives, which aligns with the findings of previous studies [20,38]. Even in the immaterial form of personal feedback, the rewards improved the compliance rate in case 3. In addition to annotations provided by participants, it is worthwhile exploring modern AI-based tools for labeling large amounts of data, such as video data.

Regarding data analysis and interpretation, data quality impacts the success of learning and adaptive applications. However, real-world sensor-based measurements often produce imperfect output data. This implies that while already advanced, sensing technology still needs to improve reliability and accuracy for field study purposes. Tools for data quality inspection (eg, the Agile Data Engine [49]) would be valuable in research-oriented field studies, but budget and resource constraints hinder the implementation of comprehensive data quality management tools. Furthermore, the influence of study participants on data amount and quality is inevitable because wearing measurement devices and providing annotations is usually required. When creating models to make decisions or predictions about human behavior based on limited datasets, it is essential to consider the generalizability and biases of the models, for instance, using existing frameworks and guidelines [37].

Our study confirmed the importance of stakeholder engagement, especially for field trials of extensive duration [16,17,25], and demonstrated the utility of the coding framework in systematically addressing this and other critical challenges. The framework not only structures the identification and management of challenges but also facilitates the comparison of results across different studies, thereby contributing to the development of more cohesive knowledge in this field.

Compared with earlier studies, our study revealed 2 new stakeholder perspectives. First, the challenge in engaging with diverse stakeholder groups with varying expectations and concerns. Second, the presence of stakeholders who are not in direct contact with the research team and who have no vested interest in the study yet whose work, responsibilities or sphere of operation is somehow affected or involved. The former requires careful handling, especially if groups have conflicting or opposing desires regarding the study outcomes. The latter requires additional effort to identify, as they may not be considered relevant by the study organization, even though they can impact the study on a practical level.

During the planning phase, it is essential to identify the actors and assess their importance and needs. One possible approach is to conduct a survey to determine the expectations and worries of each group. Stakeholder mapping is a valuable tool for assessing the significance and relevance of each group, thus enabling a better understanding of where to invest resources. It allows for a clear identification of stakeholders who have influential positions and can directly impact the implementation of the study. On the other hand, stakeholders positioned toward the outer edges of the circle can either provide support or pose potential obstacles to the research, but they do not stop it entirely. After a successful stakeholder assessment phase, active stakeholder engagement is needed.

The stakeholders need to be informed about how the research meets their expectations and manages their responsibilities in the process. The critical stakeholders must especially commit to collaborating with the researchers. However, it can be challenging to satisfy all expectations, given the experimental nature of field research. Moreover, identifying all stakeholder expectations and concerns can be tricky because their knowledge may gradually develop over time. Thus, the study organizers can ensure that the outcomes benefit all parties by implementing communication strategies that consider the distinctive needs of stakeholder groups, keep them informed, and ensure their active involvement throughout the process. The communication should also include sharing the study results with the interested stakeholders and collecting feedback to improve the stakeholder engagement process in future studies.

In conclusion, based on the mitigations and lessons from the field studies and focusing on the issues relevant to the actual research rather than project management, we provide recommendations (Textbox 1) to be considered in planning and managing a real-world study setup when collecting human behavioral data, primarily through sensors but also including self-reported material.

**Summary of the lessons learned.**

**Study setting and plan**

1. Prepare for changes in the conditions and environment with flexibility in the schedule and budget.

2. Define and focus on the main objective and ensure your study design choices support it.

3. Select measurement methods and approaches to balance between accuracy and reliability versus field study feasibility.

4. Plan for a sufficiently long field test to detect issues and phenomena.

5. Check as early as possible if you can capture the phenomenon with the data from the field study (fail fast).

6. Make user-provided annotations (ecological momentary assessments) with reminders effortless for the participants.

7. Plan for dropouts by increasing the number of participants and offering incentives (financial or immaterial rewards) to improve compliance rate.

**System and data engineering aspects**

8. Assure resources for maintaining the technical setup.

9. Conduct sufficiently long field tests to identify technical problems in advance.

10. Apply reliable tools for regular data inspection and quality assurance.

11. Understand the generalizability limitations and potential biases when creating human behavior models based on data collected from real-life settings.

**Stakeholder management**

12. Identify all possible stakeholders and assess their importance and varying needs, for instance, with a stakeholder mapping tool.

13. Identify and prepare to manage external stakeholders not in direct contact with the research team, such as maintenance, security, management, or other administrative personnel of involved organizations.

14. Recognize the most impactful stakeholders and assure their commitment.

15. Inform the stakeholders in advance how the research meets their expectations and manages their concerns and responsibilities.

16. Tailor communication strategies to different stakeholder groups, keep them informed, and ensure their active involvement.

17. Include sharing the results after the study with the interested stakeholders and collecting feedback.

### Limitations

While this study provided practical contributions to tackle challenges in longitudinal field trials monitoring humans and the environment using pervasive technology, it is essential to note some limitations. First, the purpose of the established coding framework was primarily to provide a practical structure for identifying and mitigating potential challenges that may arise when conducting long-term technology-oriented research in natural settings. As such, it can be considered a tool for field study risk management. Moreover, our stakeholder evaluation criteria were constrained to serve the scope of analysis. Thus, the framework and especially the stakeholder evaluation would benefit from further study. Second, our cross-analysis was qualitative, which is always vulnerable to subjective bias. Nonetheless, our framework and cross-study analysis provide valuable information to consider while designing and conducting a field trial for sensor-based human behavior or activity monitoring. Our future research will focus on enhancing and applying the coding framework more widely for planning longitudinal sensor-based field trials in various domains and identifying and mitigating possible challenges.

### Conclusions

Field trials are a practical method for studying human behavior and the environment and evaluating various aspects of technology in uncontrolled situations. Nevertheless, conducting field studies can be challenging, as unforeseen difficulties can occur due to the complexity of human behavior, changing contexts, and technological factors. This study introduced a novel qualitative coding framework that provides structured insights into the challenges and lessons learned from 3 field studies monitoring stress, accident-prone work activities, and environmental factors in natural settings.

According to our results, managing participants and other stakeholders, especially those interacting via a proxy or with contradictory expectations and various concerns, can be challenging. Our results imply that field trials should not only put additional effort to identify stakeholders and clarify their expectations and concerns already in the design phase but also communicate with stakeholders throughout the study process. Continuously monitoring and mitigating the possible challenges will ultimately lead to better success in longitudinal trials, improved data quality, and future technology-based solutions. Our analysis highlighted that field trials involving sensor-based human behavior assessment face unique challenges that require careful planning, robust technical solutions, and effective stakeholder engagement. By focusing on broader activity categories, maintaining flexibility, and ensuring continuous communication with stakeholders, future research can better navigate these challenges and enhance the success of longitudinal field trials.

## Abbreviations

EMA: ecological momentary assessment
GDPR: General Data Protection Regulation
HR: human resource
IEQ: indoor environmental quality
IoT: Internet of Things

## Data Availability

The data used in the study cannot be openly published because of clauses on project consortium agreements and informed consent.
